# Modeling Soil Carbon Dynamics in Northern Forests: Effects of Spatial and Temporal Aggregation of Climatic Input Data

**DOI:** 10.1371/journal.pone.0149902

**Published:** 2016-02-22

**Authors:** Lise Dalsgaard, Rasmus Astrup, Clara Antón-Fernández, Signe Kynding Borgen, Johannes Breidenbach, Holger Lange, Aleksi Lehtonen, Jari Liski

**Affiliations:** 1 Norwegian Institute of Bioeconomy Research (NIBIO), Ås, Norway; 2 Natural Resources Institute Finland (LUKE), Helsinki, Finland; 3 Finnish Environment Institute (SYKE), Helsinki, Finland; DOE Pacific Northwest National Laboratory, UNITED STATES

## Abstract

Boreal forests contain 30% of the global forest carbon with the majority residing in soils. While challenging to quantify, soil carbon changes comprise a significant, and potentially increasing, part of the terrestrial carbon cycle. Thus, their estimation is important when designing forest-based climate change mitigation strategies and soil carbon change estimates are required for the reporting of greenhouse gas emissions. Organic matter decomposition varies with climate in complex nonlinear ways, rendering data aggregation nontrivial. Here, we explored the effects of temporal and spatial aggregation of climatic and litter input data on regional estimates of soil organic carbon stocks and changes for upland forests. We used the soil carbon and decomposition model Yasso07 with input from the Norwegian National Forest Inventory (11275 plots, 1960–2012). Estimates were produced at three spatial and three temporal scales. Results showed that a national level average soil carbon stock estimate varied by 10% depending on the applied spatial and temporal scale of aggregation. Higher stocks were found when applying plot-level input compared to country-level input and when long-term climate was used as compared to annual or 5-year mean values. A national level estimate for soil carbon change was similar across spatial scales, but was considerably (60–70%) lower when applying annual or 5-year mean climate compared to long-term mean climate reflecting the recent climatic changes in Norway. This was particularly evident for the forest-dominated districts in the southeastern and central parts of Norway and in the far north. We concluded that the sensitivity of model estimates to spatial aggregation will depend on the region of interest. Further, that using long-term climate averages during periods with strong climatic trends results in large differences in soil carbon estimates. The largest differences in this study were observed in central and northern regions with strongly increasing temperatures.

## Introduction

Boreal forests act as a large sink for atmospheric carbon and contain about 30% of the global forest carbon, with the majority residing in soils as soil organic matter [1]. On upland boreal forest sites, soil organic carbon comprise 45–70% of total forest carbon [2–4]. The current sink strength has been estimated at 15% of the total forest carbon sequestration in Europe, [5] with model studies indicating that northern boreal forests may experience a loss in soil carbon [6]. Changes in soil organic carbon result from the balance between inputs and the decomposition of organic matter. While climate is an important driver for input rates as well as for decomposition, they do not necessarily show similar climate sensitivity. Both in a global and a regional context, the changes in soil organic carbon are a significant part of the terrestrial carbon cycle and concerns exist about the possibilities of increased emission rates from boreal forest soils given climate change [7, 8].

Change rates for soil organic carbon in boreal forest are relatively small, while the total pool is large, and changes result from the two opposite and large fluxes of input and decomposition. These characteristics make it challenging to reliably measure and model changes in soil organic carbon [9, 10]. Still, estimating and predicting current and future soil related carbon fluxes are central aspects of designing forest-based climate change mitigation strategies and are required when reporting to the United Nations Framework Convention on Climate Change (UNFCCC) and for forest management activities for the Kyoto Protocol. Hence significant work is ongoing in modeling soil organic carbon dynamics, with detailed small scale models [11, 12] to large scale regional [13–16] and global models [10, 17–19]. The soil carbon and decomposition model Yasso [20] and its successor Yasso07 [21–23] operate through climate driven decomposition with parameterization based on global litter mass loss data, and thus, no site- or case specific calibration is required. The organic chemical quality of litter and the dimension of woody litter are accounted for explicitly. The models were constructed to be easily compatible with forest data on large geographical scales. Yasso and Yasso07 have been applied in national reporting for the UNFCCC and the Kyoto protocol [24–28] and in local [29], regional [30–32], and global studies [19, 33] of forest soil carbon.

Forest dynamics, productivity and decomposition of soil organic matter [7] all vary across climatic gradients, and the underlying relationships to climatic variables are both complex and nonlinear, rendering up-scaling and aggregation nontrivial. This is because, generally for non-linear functions, the mean of a response variable (in our case soil organic carbon) resulting from local values of the explanatory variables differs from the response variable resulting from the mean values of the explanatory variables. Thus the aggregation level of input variables will have a consequence for the estimated soil organic carbon dynamics.

The problem of aggregation and coarse-scale descriptions of ecosystem attributes has long been known, starting with Cale et al. [34]. Further, Rastetter et al. [35] discuss four different technologies to cope with aggregation errors and how to avoid the “fallacy of averages”. They demonstrate that any nonlinear function between the smaller components will be unreliable when directly applied to the aggregate, and that the error increases with the concavity of that function and variability at fine scales. Experiences with the development of statistical models for soil organic carbon stocks show that an appropriate incorporation of carbon accumulation drivers working at different spatial scales is important [36, 37], and that model development—even with a large portfolio of explanatory variables—is challenging [38]. Statistical models for soil carbon stocks point to the importance of spatial scale considerations, but do not lend themselves to the modeling of soil carbon dynamics.

The effects of aggregating input to mechanistic models when estimating soil organic carbon stocks and changes have not been widely studied. For estimates of soil respiration, comparatively low fluxes were found when a daily flux model was used with monthly temperature data, and when temperature data were applied at the global scale compared to the grid scale [39]. However, Davi et al. [40] found only a slight bias caused by the spatial aggregation of parameters, possibly due to only moderate nonlinearities in the response functions. While these studies exemplify the issue, they are not expected to represent the effects of the nonlinearities involved in the mechanistic estimation of soil carbon stocks and changes. In the case of the Yasso07 model parameters, the response of decomposition to precipitation is highly nonlinear in a range representing a large proportion of the forest area in Norway with the mean dependence systematically lower–albeit only slightly—than the dependence based on mean climate (cf. Results section).

In regional studies [24, 27, 30, 31, 41] and in national reporting of soil organic carbon stocks and stock changes [42], it is common that climatic and forest litter input data are aggregated over time and space. Given the number of regional modeling studies of soil organic carbon dynamics, surprisingly little effort has been made to investigate the effects of temporal and spatial aggregation of input variables.

In this study, we explored the effects of temporal and spatial aggregation of climatic and litter input data on the regional prediction of soil organic carbon stocks and changes using Yasso07. When referring to forest soil organic carbon, we use the term “soil carbon” since inorganic carbon in soils was not considered. We did this through a Norway-based case study, where a) forest litter and climatic input aggregation was varied across spatial scales from the country to the regional and the plot-level and b) climatic input aggregation was varied over temporal scales between the recent long-term mean (18 years) to 5-year- and annual means. Firstly, given the nonlinear climate sensitivity in Yasso07, we hypothesized that national scale soil carbon stocks and accumulation rates would be higher when using spatially non-aggregated climate and litter input data, than when using spatially aggregated input data. Secondly, due to the observed trend with both increasing temperature and precipitation over the past decades, we hypothesized that using historical long-term mean climate would lead to higher estimates of soil carbon stocks and stock changes.

## Materials and Methods

### The national forest inventory

**Fig 1:**
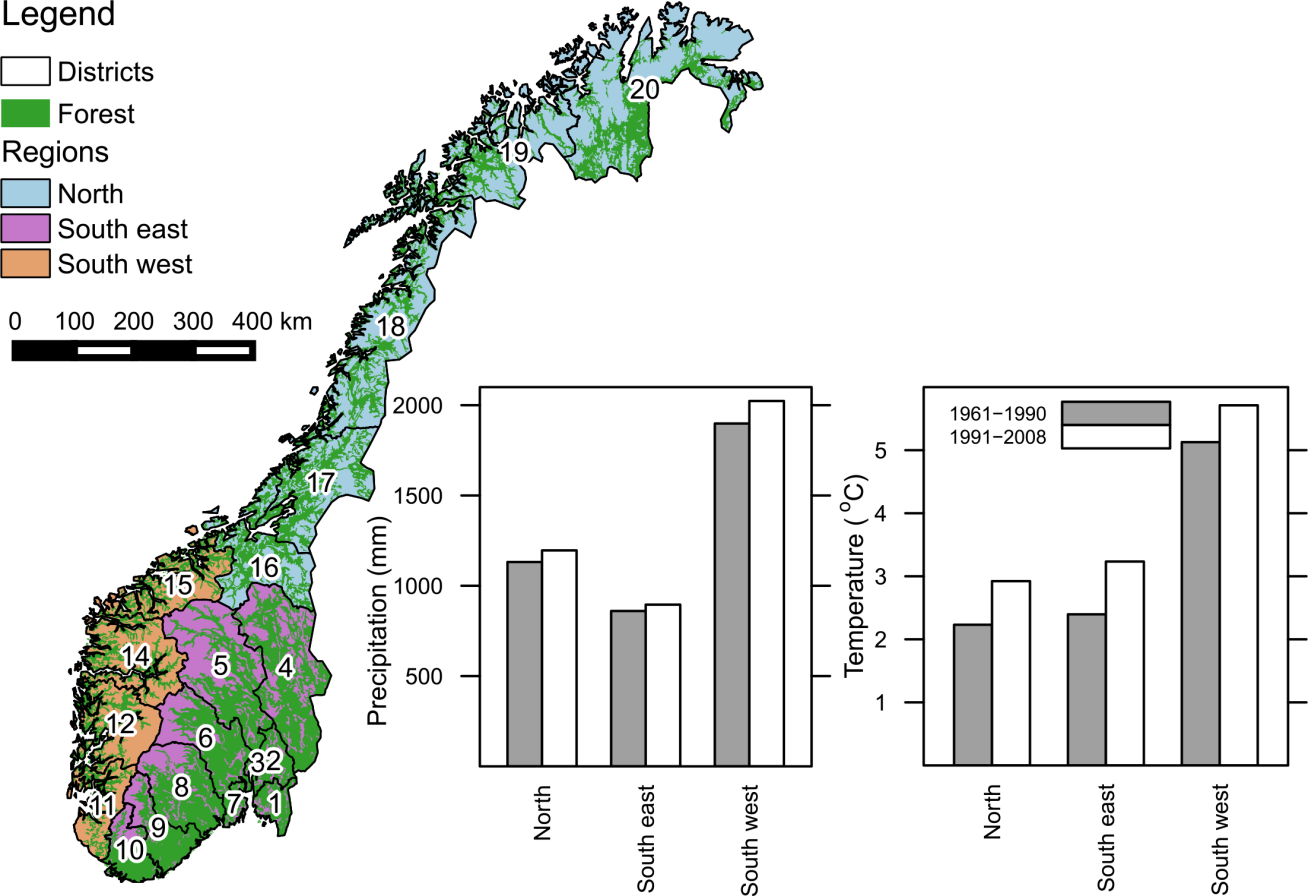
Forest area and regional climate in Norway. The forest is distributed throughout the country, but the majority is found in the southeastern and central parts (left panel). Mean annual temperature and mean annual precipitation in the three regions indicate a warmer and slightly wetter climate more recently than prior to 1990.

The basic structure of the national forest inventory (NFI) is a 3×3 km grid of circular permanent sample plots (radius 8.92 m) covering Norway [43, 44]. The majority of the current permanent sample plots were established in1986-1993 but the NFI has been carried out regularly since 1919 using varying designs. In Finnmark (district 20, the northernmost district of the country, Fig 1) and in areas above the coniferous forest limit, permanent sample plots were established 2005-2009/2010 and were sampled using a larger grid-size; 9×9 and 3×9 km, respectively. Stand and tree characteristics are quantified on each NFI plot every 5 years, i.e. the whole country is fully covered each 5 years. The NFI represents the full range of stand productivities, developmental stages, and climatic conditions in Norway (Fig 1), where the southwest region stands out as relatively warm and wet. In this study, we used the time series for the NFI as applied for UNFCCC reporting. This implies that minor inconsistencies among consecutive inventory intervals have been adjusted. In addition, the state of plots above the coniferous forest limit and in Finnmark back to 1990 has been estimated using tables of average biomass change for different forest types stratified according to site productivity, age, and species composition. These tables were developed using data from the 8^th^ (2000–2004) and 9^th^ (2005–2009) inventory [45]. In this study, a total of 11275 NFI plots were used in simulations.

### The litter decomposition and soil model

**Fig 2:**
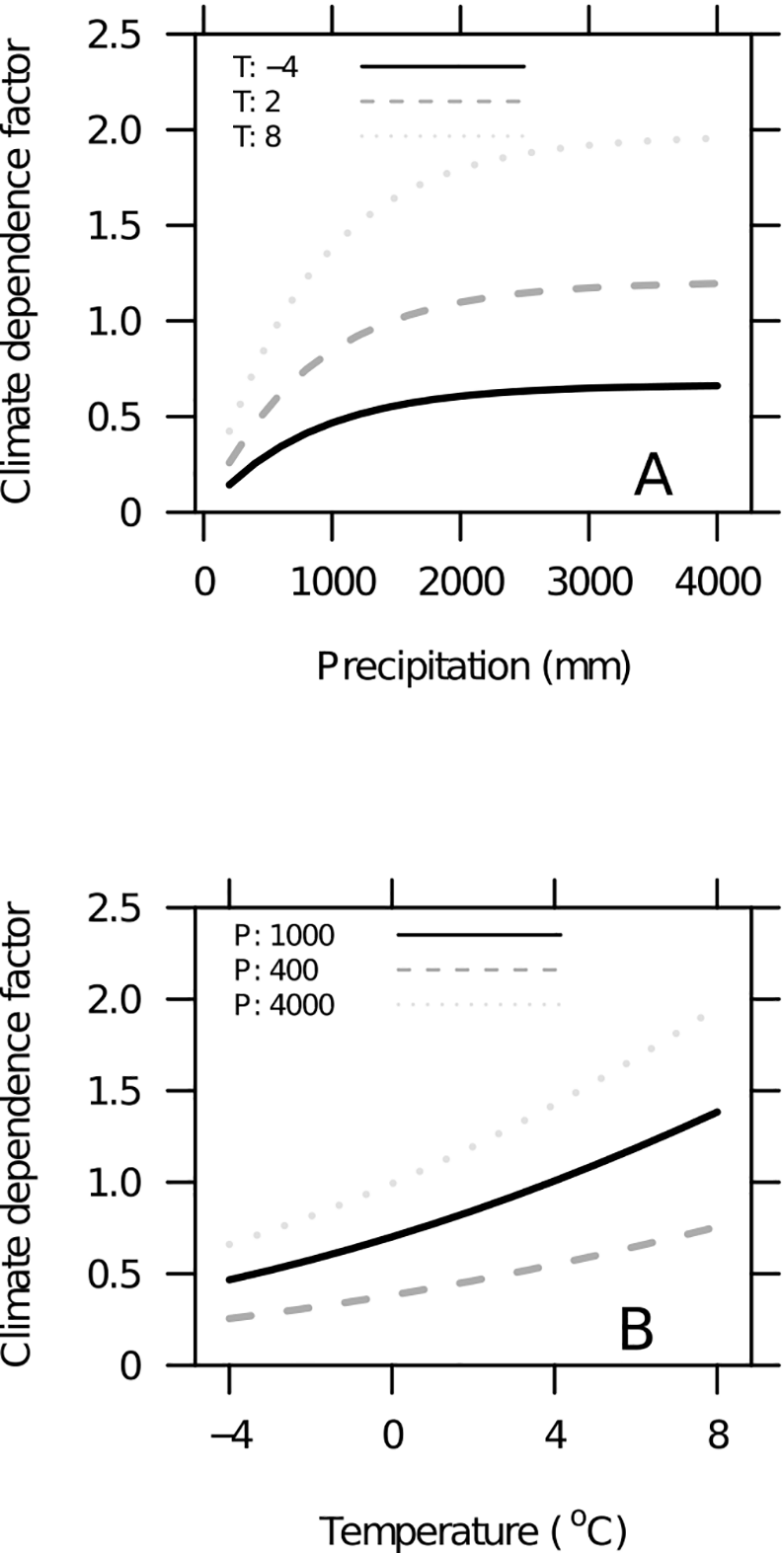
Climate dependence factors for decomposition in Yasso07 representing the climatic range in Norway. (A) Dependence factors (Eq 1) for three fixed values of mean annual temperature, T (-4, 2 and 8°C, respectively). At constant T, dependence factors increase with increasing precipitation up to a finite asymptotic value and become practically independent from P once it exceeds 1500 mm per year. (B) Dependence factors (Eq 1) for three fixed values of precipitation, P (400, 1000, 4000 mm, respectively).

Yasso07 is a generalization of Yasso [20] based on three assumptions regarding the litter decomposition process [21–23]. Firstly, it assumes that litter consists of four different chemical compound groups called the labile groups, each with a mass loss rate independent of the origin of the litter. These groups are E: soluble in a non-polar solvent (ethanol or dichloromethane), W: soluble in water, A: hydrolyzeable in acid or N: neither soluble nor hydrolyzeable. Secondly, the mass loss rate of each compound group depends on precipitation and temperature. The climate dependence of decomposition is assumed in the model to be identical for all compound groups as it proved impossible to parameterize for each compound group separately [23]. The climate dependence of the decomposition rates (k_*i*_) is formulated as:

$$k_{i}(C)=\alpha_{i}\text{exp}(\beta_{1}T+\beta_{2}T^{2})(1-exp[\gamma P_{a}])$$ (1)

where T is temperature (°C), P_a_ is the annual precipitation (m), *α*_*i*_ is the reference decomposition rate at T = 0°C for the compound groups, *β*_1_, *β*_2_ and *γ* are model parameters and *i* indicates the compound group in question [23]. T is calculated from mean annual temperature and the lowest and highest monthly temperatures using a sinusoid to account for intra-annual temperature variations. The climate dependence modifies the reference decomposition rates in a nonlinear manner, particularly for precipitation lower than 2000 mm (Fig 2). Thirdly, decomposition results in the formation of a more recalcitrant compound group (humus, H), the end product of mass flow among the compound groups, and mass loss from the system. The decomposition of woody litter in the model depends on the dimension. Thus, large elements like tree stems and stumps will go through a process specific for woody litter decomposition in addition to the process of non-woody litter decomposition. The full decomposition of large elements will take place over a longer time span relative to small elements [21]. Litter dimension is accounted for using three size classes where for non-woody litter (bark, foliage and fine roots for trees, ground vegetation litter) the dimension is 0 cm. A dimension of 0 cm reflects that no features specific for woody litter are applied. For fine woody litter (branches and coarse roots from trees) and coarse woody litter from natural mortality and harvest (stems and stumps), dimensions are 2 cm and 10 cm, respectively, indicating an increasing importance of the woody-litter-specific decomposition features. The data used for model parameterization consists of mass loss measurements (3–10 years) from litter bag studies on 97 sites in Europe, North America, and Central America. Leaf litter from 34 different species is included. In addition, soil carbon stock measurements from 26 sites in Finland from a 5500-year soil chronosequence are included [46]. Decomposition measurements (n = 2102) of woody litter [21] are used from four boreal forest sites and include litter of Norway spruce (*Picea abies* (L.) Karst.), Scots pine (*Pinus sylvestris* L.) and birch (*Betula pendula* Roth. and *Betula pubecens* Ehr.), with litter size ranging from 0.5 to 60 cm and decomposition followed for 1–70 years since the start of decomposition. Yasso07 comprises a set of first order differential equations and the flows among compound groups were unconstrained when fitting the parameters to data. Parameter values (the maximum a posteriori point estimate and the 95% confidence set for the model parameter vector) are estimated using a Markov chain Monte Carlo technique. Yasso07 is written in the fortran programming language and the code is available at (https://code.google.com/p/yasso07ui/). Software and statistical procedures used in the parameterization of Yasso07, including parameter uncertainties, are described by Tuomi et al. [22, 23]. Yasso07 represents the organic soil carbon down to 1 m depth but with no vertical allocation of the carbon pools (i.e. the total in mineral soil and in the organic layers but without representation of soil horizons).

### Litter input from trees and ground vegetation

The annual litter input was estimated for five different litter input sources which were: i) the living trees on each NFI plot (Norway spruce, Scots pine and birch/deciduous); ii) ground vegetation (lichens, moss, shrubs, herbs and grasses); iii) natural mortality; iv) residues from non-commercial wood extraction and v) residues from commercial thinning and final harvest. The sources i) and v) were based on tree registrations on individual NFI plots and ii), iii), and iv) were based on models using NFI plot variables such as site index, stand age, and dominant tree species as input. Once estimated, the chemical litter quality (the distribution to compound groups) for the litter components was based on a global database for tree litter chemistry [23, 44] and for ground vegetation [47]. Further details on the estimation procedure for each of the five sources are: i) Individual tree biomass was estimated from models with tree height and breast height diameter (stem diameter at 1.3 m tree height) as input variables [48, 49]. Birch foliage was estimated using a relationship to stem biomass [44, 50], and birch stump biomass was estimated using the pine stump biomass model due to a lack of specific data and models for birch. Fine root biomass for all tree species (root diameter < 2 mm) was estimated as 0.3 × foliage biomass [51]. ii) Above ground biomass in ground vegetation was estimated from dominant tree species and stand age [52, 53]. Below ground biomass in ground vegetation was assumed to be twice the amount of above ground biomass as indicated by studies compiled by Peltoniemi et al. [47]. Turnover rates were used to estimate litter from living vegetation (Tables 1 and 2). iii—iv): Tree litter from natural mortality and residue from non-commercial wood extraction was estimated as a percentage of the living tree biomass based on tables of average biomass change as described above; [45]. v): harvest residues from commercial thinning and final harvest were estimated from plot specific registrations of harvested volume. Litter input was constant for each 5-year period.

**Table 1:** Annual turnover rates (year^-1^) applied for the estimation of tree litter input.

Component	Norway spruce	Scots pine	Deciduous	Reference
Foliage	0.143	0.33	1	[54]
Live and dead branches, Roots > 2 mm	0.0125	0.027	0.025	[55],[56],[57]
Fine roots < 2 mm	0.6	0.6	0.6	[58]

Compiled in [*47*] and [*31*].

**Table 2:** Annual turnover rates (year^-1^) used to estimate litter input from ground vegetation.

**Component**	**Moss**	**Lichens**	**Herbs and grasses**	**Dwarf shrubs**
Above ground	0.33	0.1	1	0.25
Below ground	-	-	0.33	0.33

Compiled in [*47*].

### Initial values for soil carbon

Initial soil carbon stocks may be given to Yasso07 directly by the user. However, its distribution to the five chemical pools (AWENH) cannot easily be estimated a priori and an unbalanced pool initialization may result in unrealistic model output. To avoid this, the model pools were initialized by a spin-up procedure running the model for 5000 years (time-steps) with constant input to estimate a steady-state condition. Litter input used in the spin-up should ideally reflect the history and litter production of the site prior to the simulation time series. To represent stand history at the scale and resolution in this study, we used information available in the first permanent sample plot NFI (1986–1993; 2005-2009/2010 for Finnmark and mountain forests) to set up a number of rules for stand development starting 35 years prior to simulation start (for individual plots, simulation start was the year of the first permanent sample plot registration; for the country as a whole the start was defined as 1990). Based on the rules for stand development, a historical litter input time series (used in a pre-simulation representing the years 1960–1990 for the country as a whole) was created. The earliest observation in the pre-simulation time series was 1956 (plots established in the field 1986). The estimate of litter input from harvest residues relied on the standing biomass 5 years earlier, hence the need for information on stand development 35 years prior to simulation start. In 76% of the plots (stand age > 34 years), this was a matter of estimating previous stand dynamics of the same stand. This was based on mean biomass development in a 5-year period between two recent NFI’s (average biomass change; as described above; cf. [45]). For young stands of unproductive forest (3% of the plots), biomass was kept constant back in time and equal to that of the first NFI registration. For young productive stands, rules were set up to reflect registrations on previous land-use: 1) continuous forest cover of the same species including knowledge of commercial harvest (ca. 19% of plots), 2) forest following agricultural land-use or change of tree species following low productive birch or pine forest (ca. 2% of plots). When relevant, aerial photos were consulted to identify the previous land-use. Above the coniferous forest limit and in Finnmark, stand development was estimated back until age zero and then kept constant further back in time until the start of the pre-simulation. These are forests of low productivity, and in reality the age-related changes for these areas are minor. Harvest residues from commercial harvest in the time prior to the first NFI registrations was based on mean standing volume estimates of NFI registrations prior to 1986 (1964–1976, 4^th^ NFI) at recommended harvest age by site index and dominant tree species. The reason for using older registrations on volume at harvest age was that standing volume was lower in the 1960’s and 1970’s than now for comparable site indices. The Yasso07 simulations were started for each plot using a spin-up to a steady-state and the 30-year means for plot climate (see below) and the mean litter input (by site index and dominant tree species) at the time of the first registrations in the permanent-plot NFI. After the spin-up, the historical litter time series for each plot prior to the first NFI registrations was used (pre-simulation) followed by the litter input time series based on the actual permanent plot NFI registrations (the current litter time series). Over the initialization period we assumed that the plots were under forest cover. Further, we assumed that the soil carbon pools were in a steady-state at the beginning of the pre-simulations (= end of initialization). However, after the pre-simulation (reflecting the years ca. 1960–1990) the soil pools were no longer in a steady-state. A similar approach was used to initialize model runs for soil carbon development in Swedish forests [32].

### Climate data

Climate for the spin-up calculation was the most recent climate normal (1961–1990) and for the litter time series (historical and current) calculations of soil carbon stock change over time (when simulating with constant climate) it was the mean for 1991–2008. Climatic data were obtained from the Norwegian Meteorological Institute and were extracted from a 1 x 1 km climate grid established by interpolation of climate station measurements across Norway [59].

### Simulations

A total of five different simulations were run (Table 3). Simulation I, the current standard used for UNFCCC reporting, used long-term climate means at the plot-scale. In addition to the standard (simulation I), two spatial aggregation levels were applied for the climatic input (Table 3): 19 administrative units (districts) or the entire country (Fig 1) where climate input was held constant over time and means (1991–2008) were computed across the relevant NFI plots on upland forest. The results from simulations with district (simulation II) or country (simulation III) aggregation levels are referred to as “effects of spatial scale” and contrasted with simulations on the plot-scale (simulation I). All simulations where spatial scale was compared were run using constant climate.

**Table 3:** List of simulations and the spatial and temporal aggregation levels applied for climatic input.

Simulation	Spatial scale	Temporal scale	Description
I	Plot	Long-term mean	Standard; currently used for UNFCCC reporting
II	District	Long-term mean	Effects of spatial scale
III	Country	Long-term mean	Effects of spatial scale
IV	Plot	5-year mean	Effects of temporal scale
V	Plot	Annual mean	Effects of temporal scale

Climate variability over time (beginning 1960 i.e. including the pre-simulation) was introduced by 5-year means (simulation IV; the mean of years between individual plot registrations was used; in a few cases this deviated from 5) or by annual climate (simulation V; using climate for the year of each individual plot registration). The results of simulations IV and V are referred to as “effects of temporal scale” where spatial scale was held constant (plot) and results were contrasted with simulations using constant climate (simulation I).

Output from the simulations was expressed in kg C m^-2^ (stocks) or kg C m^-2^ 5 years^-1^ (changes). An exception was that a national estimate for annual soil carbon stock change was obtained by multiplication with the appropriate representation factor of each NFI plot (for example, in the 3 x 3 km grid each plot represents ca. 900 ha). Thus, the total national estimate for soil carbon stock change over time was based on the representative and systematic sampling comprising the basis of the NFI [43, 44]. Model outputs shown were from the years 2008–2012 (soil carbon stocks) and 2000–2012 (soil carbon changes). For national estimates of soil carbon changes the full length of the time series was used (earliest plot-specific time series starting 1956 and last observations in 2012). The model application was limited to the NFI plots found on mineral soil which relies on routine NFI field registrations and is defined operationally as plots with an organic soil horizon thickness < 0.4 m (upland forest). Technically, we computed climate input for the spatial and temporal scales in question and applied this on each NFI plot without manipulation of litter input. The extraction of data from the NFI data base, necessary input manipulation and call to Yasso07 as well as post-processing of output was done with R version 2.11.1 ([60]).

## Results

**Fig 3:**
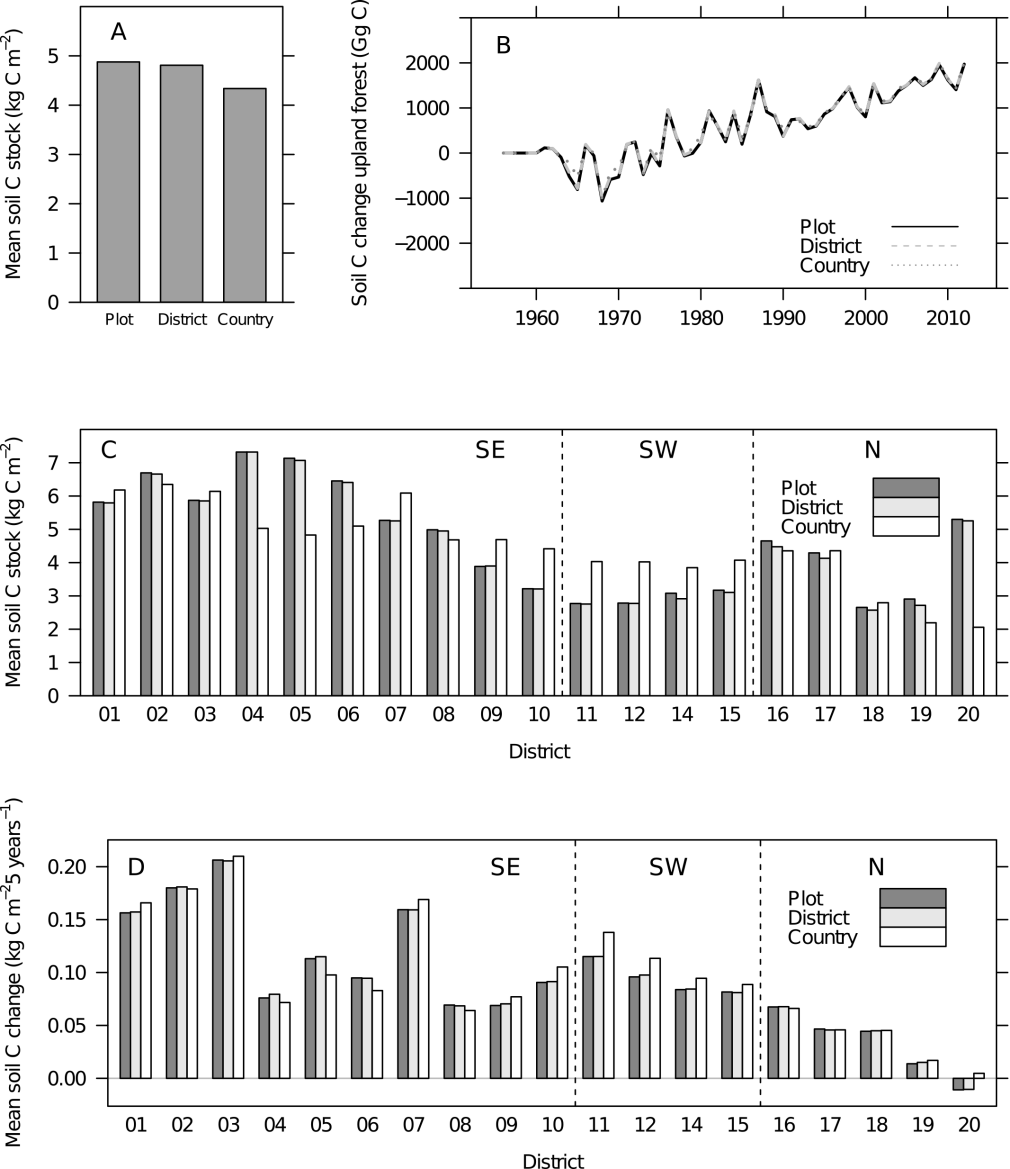
Soil carbon stocks and changes using Yasso07 and Norwegian NFI data–effects of spatial aggregation. All the simulations shown used constant mean climate for 1961–1990 and 1991–2008 for spin-up and time series calculations, respectively. Simulations differed by representing three levels of spatial aggregation: plot- (n = 11275), district- (n = 19) and country-scale. Vertical lines (figure parts C and D) indicate distribution of districts to regions southeast (SE), southwest (SW) and north (N) (see Fig 1). (A) Overall mean of estimated soil carbon (soil C) stocks (2008 to 2012) was lower for the country-scale than for plot- and district-scale. (B) Soil carbon change estimates scaled to the national level. Simulations for different aggregation levels showed almost identical results. (C) District means of estimated soil carbon stocks (2008 to 2012). When applying plot- or district-level climate, substantial variability in estimated stocks emerged. (D) District means of soil carbon stock change estimates (2000 to 2012) were similar across the three spatial aggregation levels, but varied by district.

**Fig 4:**
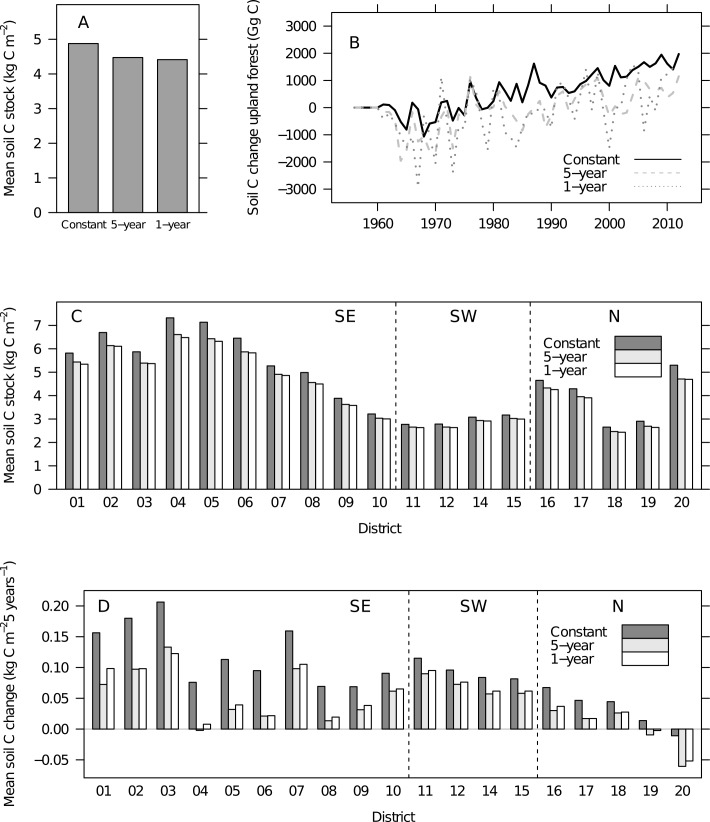
Soil carbon stocks and changes using Yasso07 and Norwegian NFI data–effects of temporal aggregation. All simulations shown were run for each of 11275 NFI plots using plot-specific climatic input including the 1961–1990 mean climatic values for spin-up. For the time series following the spin-up calculation, the simulations differed by representing three levels of temporal aggregation of climatic data: constant mean values, 5-year mean, and annual mean. Vertical lines (figure parts C and D) indicate distribution of districts to regions southeast (SE), southwest (SW) and north (N) (see Fig 1). (A) Mean of estimated soil carbon (soil C) stocks (2008 to 2012) was highest when constant mean climate was applied. (B) Soil carbon change estimates scaled to the national level. Simulations with constant climate showed higher change rates than those using a higher temporal resolution. (C) District means of estimated soil carbon stocks (2008 to 2012) were consistently higher when constant climate was used. (D) District means of estimated soil carbon stock changes (2000 to 2012) were consistently higher when constant climate was applied. Substantial differences across aggregation levels emerged particularly in the southeastern and in some northern districts.

The results illustrated that both spatial aggregation and temporal aggregation of climatic data had considerable effects on the modeled outcomes. Specifically, the spatial aggregation of climatic data was found to lower the estimated soil carbon stock with only minor effects on current soil carbon change rates (Fig 3). The use of long-term average climatic data resulted in higher soil carbon stocks and accumulation rates than when using annual or 5-year mean climatic data (Fig 4).

### The effects of spatial aggregation of climatic data

**Fig 5:**
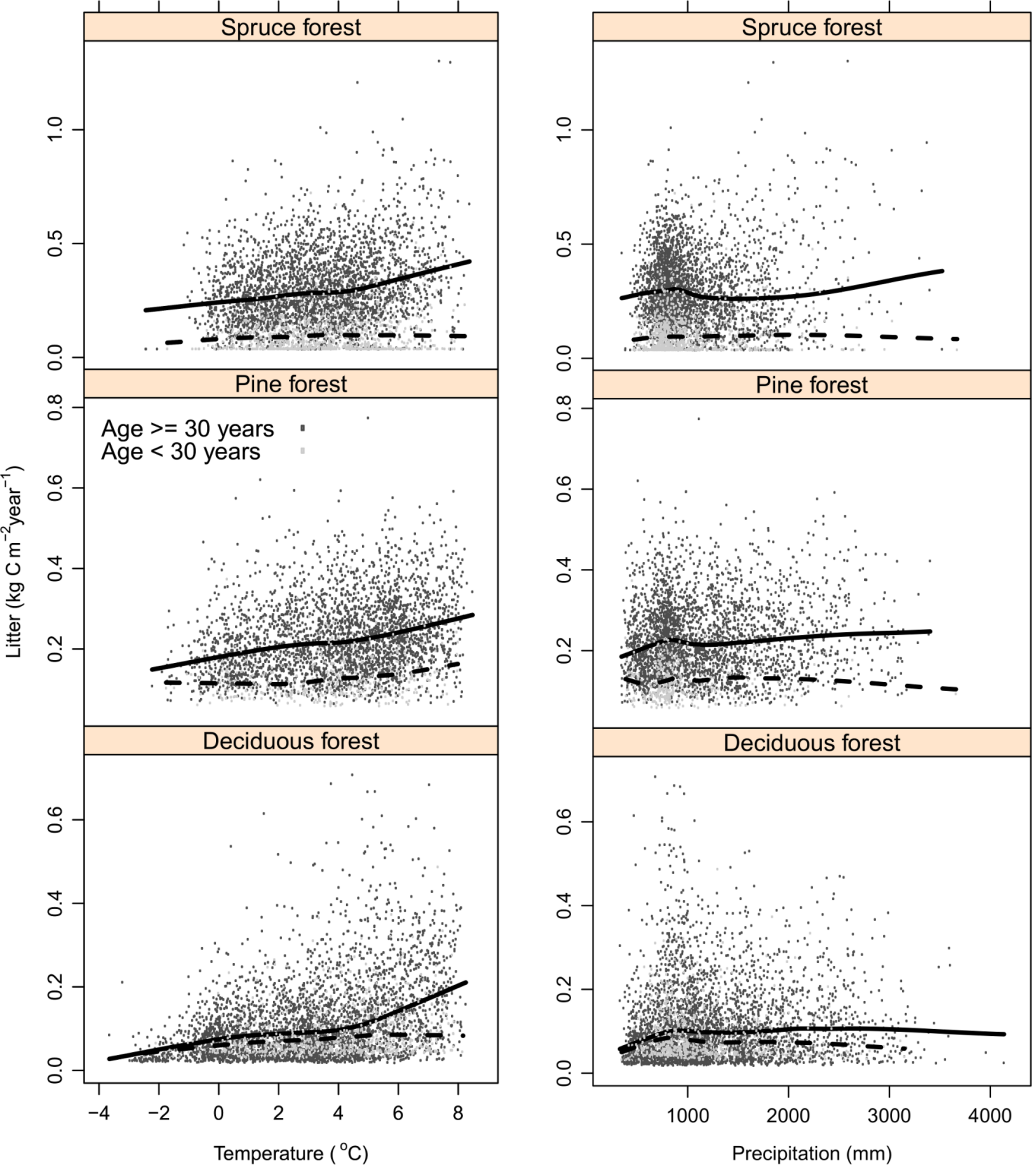
Forest litter input. Estimated litter input showed a tendency to increase with temperature regardless of dominating tree species and was highest for mature and old forest. Spruce-dominated forest showed the highest estimates of litter input. The figure includes litter from living trees, ground vegetation, and natural mortality. The estimated mean annual litter input (m^-2^) across all forest types was 0.195 kg C (carbon) (excluding harvest residues) and 0.221 kg C (including harvest residues). “Spruce forest”, “Pine forest” and “Deciduous forest” comprise plots where the respective tree species dominate. Lines are standard smooth functions in R (type = smooth, span = 0.5) for mature and old forest (full line) and young forest (broken line).

The calculated plot-level litter input data showed large inter-plot variability with apparent trends between species, climate and input quantities (Fig 5). The observed trends illustrated that spruce forest had higher input rates than pine and deciduous forests and that input rates were higher in mature and old forest relative to young forests (Fig 5).

The national mean forest soil carbon stock estimate was about 10% lower when using country-level aggregated input data compared to plot- or district-level input data (Fig 3A). Regional trends became obvious for the soil carbon stock estimates across the 19 districts when estimates with country-level input data were compared to estimates with plot- or district-level input data (Fig 3C). In the coastal districts (districts 9–15, Fig 1), that have the warmest and wettest climate, using country-level input data led to considerably higher stocks compared to when plot- or district-level climatic data was used (Fig 3C). The opposite was observed for the colder and drier districts in the east and the north (Fig 3C).

The national total forest soil carbon stock change estimate was barely influenced by the level of spatial aggregation (Fig 3B). When comparing the soil carbon stock change estimates for the 19 districts using the country-level input data compared to plot- or district-level data, similar but less pronounced regional trends as for soil carbon stocks became apparent (Fig 3D). In the coastal districts, the use of country-level input data led to the highest estimated accumulation rates but for the other regions, results were similar across simulations and in the colder and drier districts country-level input data tended to produce the lowest accumulation rates (e.g. district 4–6; central mountains). Interestingly, using plot- or district-level input data resulted in an estimated loss of soil carbon in the northernmost district (Fig 1).

**Fig 6:**
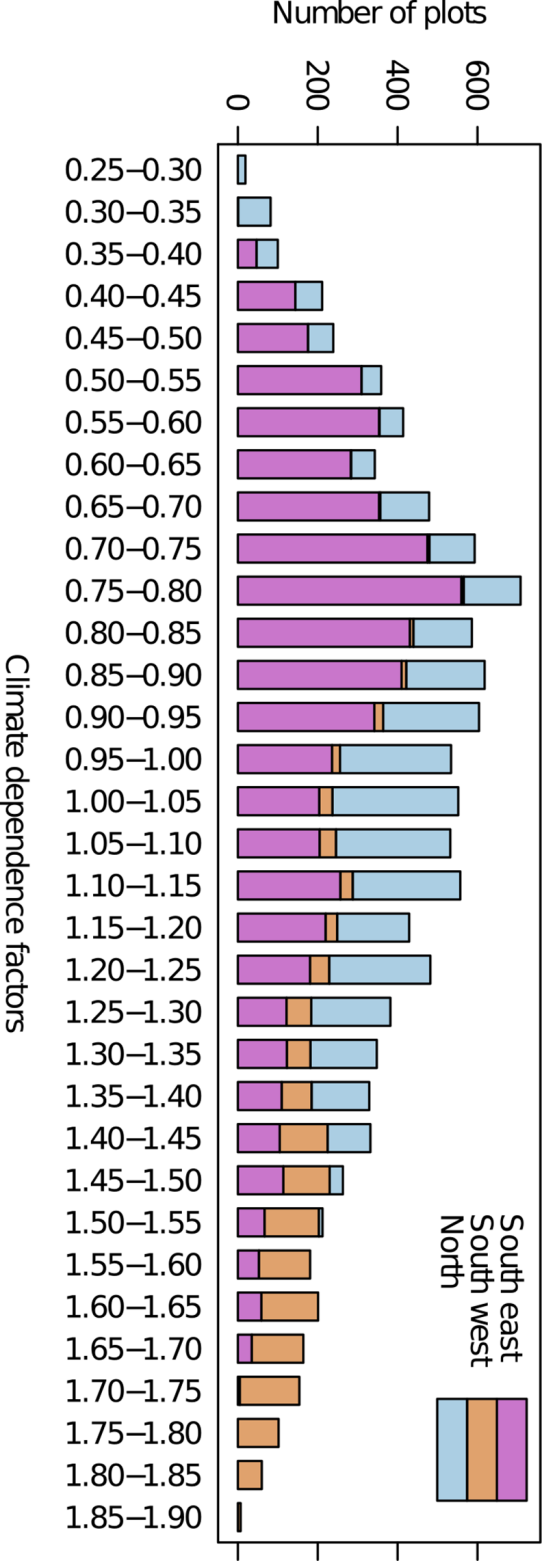
Frequency distribution of Yasso07 climate dependence factors. Dependence factors were generated for each NFI plot using constant climate (long-term mean 1991–2008), and colors show their distribution to three regions (regions are shown in Fig 1). The dependence factors were generally highest in the southwest and lowest in the southeast.

In Yasso07, decomposition is driven by climate dependence factors which showed a large variability when calculated with plot-level long-term mean climatic data (Fig 6). Climate dependence factor values (Eq 1) for plot-scale and constant climate ranged from 0.2 to 1.9 and caused an 80% reduction to 90% increase in the reference decomposition parameters, (Fig 6). The median and mean of all plot-level dependence factors was 0.972 and 1.004, respectively. A slightly higher climate dependence factor (1.031) was found when using the country mean temperature (3.5°C) and precipitation (1150 mm). Generally the lower and intermediate climate dependence factors were prominent (Fig 6) in the southeastern and northern region (Fig 1), while the high climate dependence factors were found primarily in the southwestern region.

### The effects of temporal aggregation of climatic data

**Fig 7:**
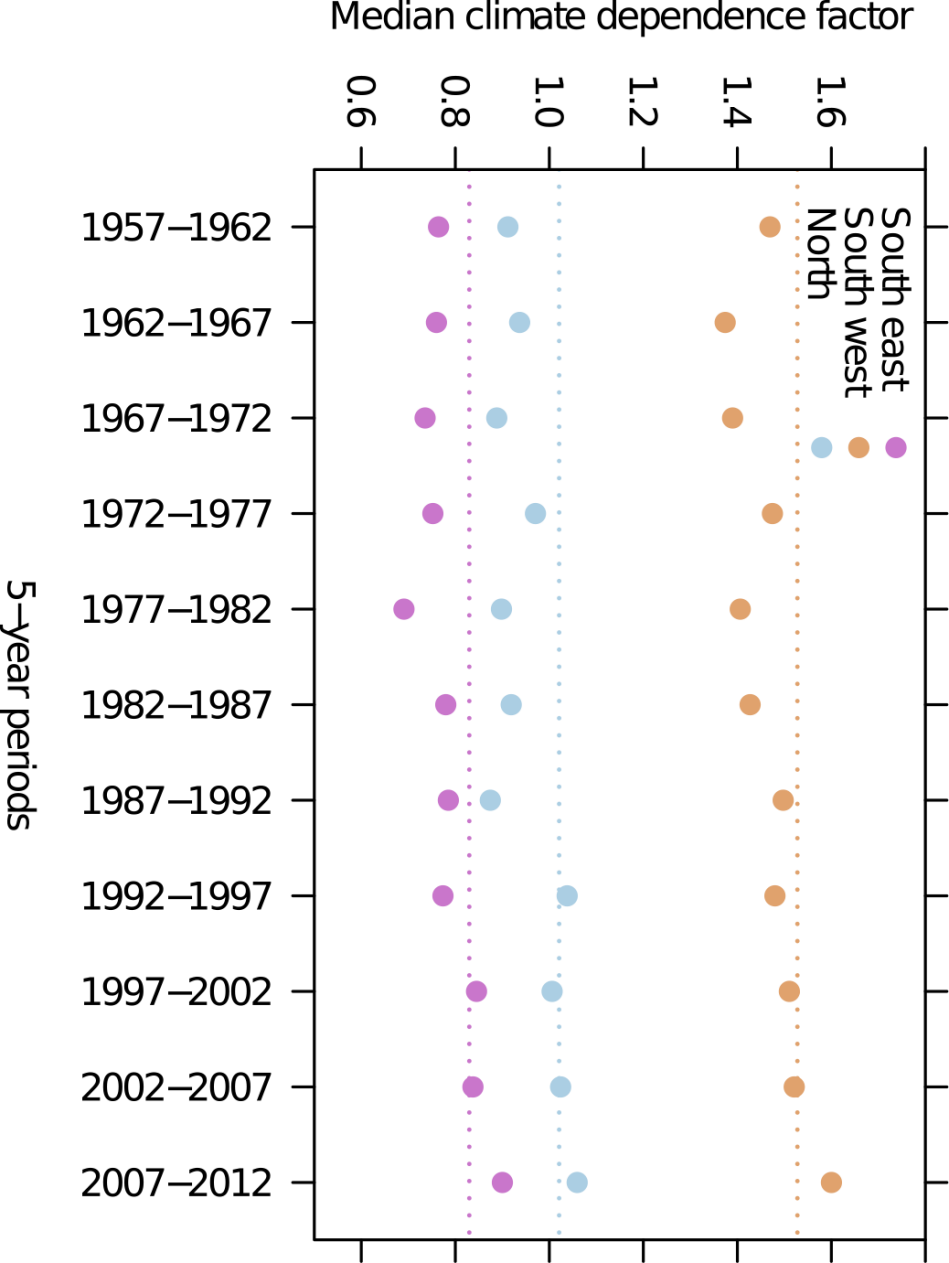
Regional climate dependence factors over time. All three regions showed an increase in climate dependence factors since 1960 (medians by 5-year periods, based on 5-year mean climate). The broken lines indicate, for each region, the median climate dependence factor using constant plot climate 1991–2008.

The national mean forest soil carbon stock estimate (Fig 4A) and the national total soil carbon change estimates in particular (Fig 4B) were strongly affected by the temporal aggregation of climatic data. Using long-term climate means led to higher mean soil carbon stocks than when using annual or 5-year aggregated data. When comparing the soil carbon stock estimates for the 19 districts estimated with long-term aggregated climatic data and annual or 5-year climatic data (Fig 4C), regional trends became apparent. In coastal districts, (Fig 1) the use of long-term averages led to slightly higher or similar soil carbon stocks as compared to 5-year or annual climatic data. The coastal districts are the warmest and wettest region of Norway and have had, in relative terms, less warming and less of an increase in precipitation than other parts of the country (Fig 1). Simultaneously, the climate dependence factors in this region were generally high (Fig 6), and were of the magnitude where the function for estimating the dependence factor is asymptotic for precipitation (Fig 2). On the other hand, in the colder northern and southeastern regions (Fig 1), the use of long-term climatic data led to considerably higher stock estimates as compared to 5-year or annual climatic data (Fig 4C). In the southeastern and northern regions with a significant increase in temperatures during recent years compared to the long-term mean (Fig 1), the climate dependence factors were relatively low (Fig 6). Thus, in the southeastern and northern regions, dependence factors were in the range where they are sensitive to variations in both temperature and precipitation (Fig 2). Climate dependence factors have increased for all regions over the last decades, but the increase was greater in northern and southeastern regions (Fig 7).

**Fig 8:**
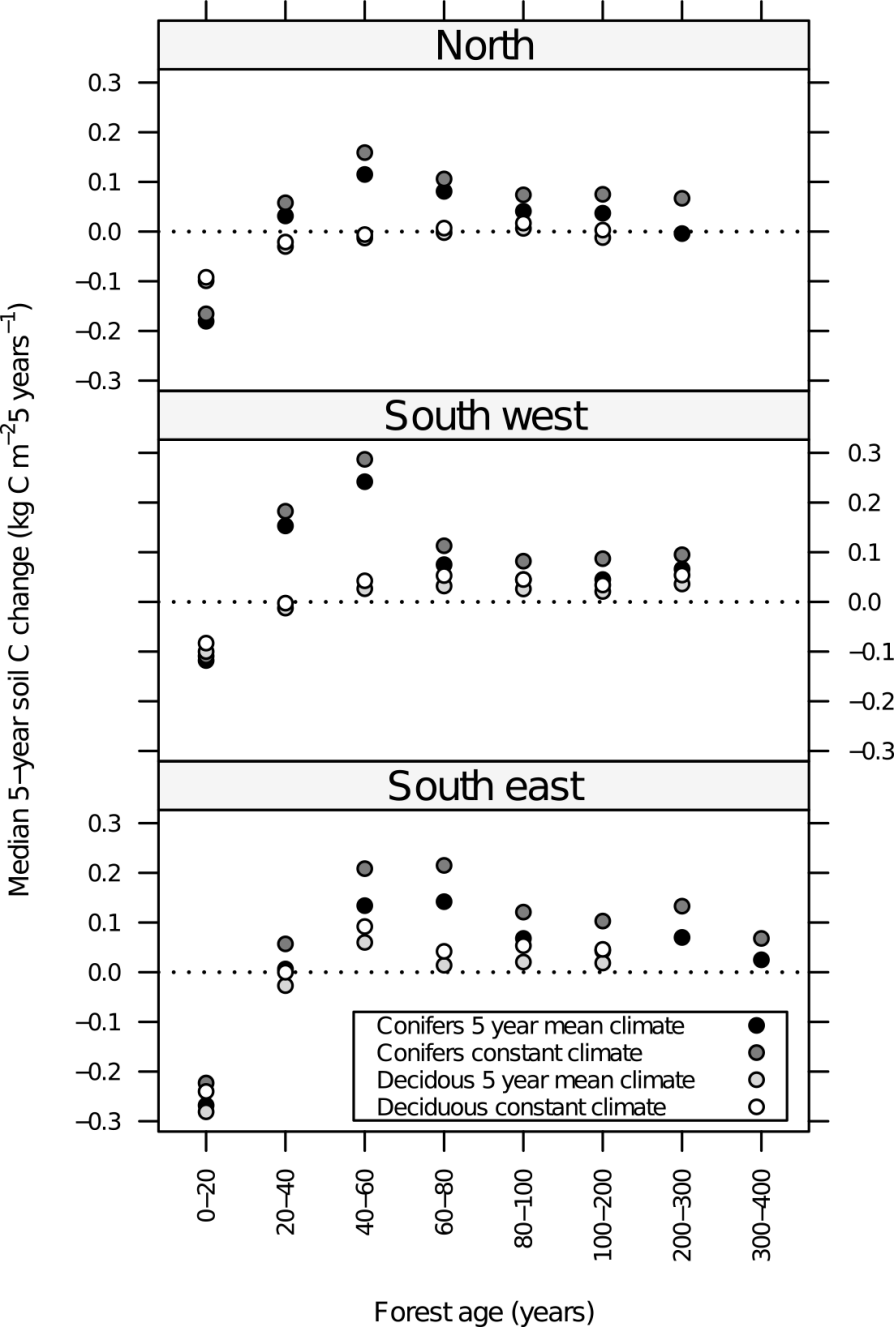
Yasso07 predicted soil carbon change rates across groups of age-class, dominant tree species, and region. Soil carbon (soil C) change rates were given as the change estimated for 5 years (i.e. the time between NFI registrations on a plot) and aggregated as the median value in each age-class group. Simulations used constant plot climate or 5-year mean climate–both at the plot-scale. The accumulation rates were higher in coniferous forest than in deciduous forest and for both forest types, accumulation rates were higher when using constant plot climate compared to that of 5-year means.

The national total forest soil carbon stock change estimate was strongly influenced by the temporal aggregation of climatic data (Fig 4B), with long-term aggregation leading to higher estimates of soil carbon accumulation rates than using annual climatic data. The annual mean national total forest soil carbon change (2000–2012) was 60–70% lower when annual or 5-year averages of input data was applied compared to the estimates using constant climatic input (450–570 compared to 1480 Gg C). When comparing the soil carbon stock change estimates for the 19 districts (Fig 4D) with variable temporal scale input, strong effects emerged: In the coastal districts the use of long-term mean input data led to the highest estimated accumulation rates though the variation among simulations was moderate compared to the other regions. In the eastern and northern regions (Fig 1), the use of long-term input data resulted in accumulation rates more than twice as large as when using annual input data (Fig 4D). The higher estimated accumulation rates with the use of long-term constant climate were consistent across all tree species and age-classes (Fig 8).

## Discussion

Simulations for stocks and stock changes of forest soil carbon showed that a country-level average soil carbon stock estimate may vary by 10% depending on the spatial and temporal scale of input data. Further, the estimated country-level stock changes were considerably lower with annual or 5-year mean climatic input data as compared to estimates using constant climate, reflecting the dynamics in the recent climatic changes observed for Norway.

### Spatial scale

We hypothesized that higher soil carbon stocks would result from a disaggregated estimation procedure than from a spatially aggregated procedure which was supported by our results showing a 10% higher estimated stock than the aggregated approach. Knowing that the soil carbon stock represents a large part of the total forest carbon, even this moderate deviation represents a large amount of carbon. The spatial effects were driven primarily by representing the local climate in the relatively dry and cold central and some northern districts with a high forest cover.

Contrary to our hypothesis, soil carbon changes were not sensitive to the level of spatial aggregation. The soil carbon stocks primarily result from the spin-up procedure using the mean litter input rate based on forest type. In spite of only small effects on change rates from the spatial level of climatic input, these effects accumulate during the spin-up time. Thus, any spatial aggregation effect on the annual change rates may be comparatively low. In addition, the 50 years of contemporary simulation would be influenced by other processes on the short-term time horizon such as differences among inventory rotations and forest age distribution.

The differences among spatial scales for estimated soil carbon change rates across districts were small. For stocks as well as for stock changes, the plot-scale and the district-scale produced very similar results indicating that results were not sensitive to the scale of input data on the plot- and district-level. Using the fine spatial scale, represented by the plot-level, could be beneficial for other reasons e.g. providing data to local carbon balance estimates, which are increasingly requested by local and regional stakeholders. Further, the plot-level application makes it possible to evaluate results on estimated soil carbon dynamics relative to other NFI measurements or registrations without any initial averaging.

The importance of spatial scale for predicting landscape level functions has been stressed, for example, for fire [61], for carbon dynamics in paddy fields [62] and for the development of statistical models for soil carbon stocks at the landscape and national scale [36, 37, 63]. In the latter, particularly, the value of incorporating information on topography, hydrology, and soil physics were found to be crucial. However, to our knowledge, little has been documented on the magnitude of spatial aggregation errors for the mechanistic modeling of soil carbon stocks or changes in forests. Spatially explicit modeling of the long-term total forest carbon sink in Canada was 21% lower than when a spatially aggregated model was used [64, 65]. Both of the Canadian model studies included recent climatic changes. In the two Canadian studies, the observed difference was argued to result from the representation of high latitude carbon dynamics, i.e., a relatively smaller response to temperature in NPP, higher response in heterotrophic respiration and a more persistent delay in regrowth after disturbance than the country mean [64]. This was supported by our estimates for the districts in the far north, where negative change estimates indicated that the representation of the forest and climate dynamics in high latitudes is important.

In this study we found that higher soil carbon stocks would result from a disaggregated estimation procedure than from a spatially aggregated procedure and we found that soil carbon changes were not sensitive to the level of spatial aggregation. However, we would argue that the differences among estimation procedures (i.e. effects of aggregation) will depend on the region of interest and that the general result from this study is that the level of aggregation will potentially influence estimated soil carbon stocks and stock changes. Hence, we would advise caution when evaluating estimates of soil carbon e.g. from studies with large-scale spatial aggregation (e.g. [30, 31, 41]).

### Temporal scale

As hypothesized initially, using historical long-term mean climate led to higher estimates of soil carbon stock changes compared to using annual or 5-year averages. These effects were strong enough to result in a ca. 8% lower country mean soil carbon stock estimate using annual climate compared with long-term mean climate input. Effects were caused primarily by an increase in temperature and climate dependence factors in the southeastern and northern districts. While soil warming experiments have generally shown a marked increase in soil respiration [66], only part of this increase is due to changes in decomposition rate. There is general agreement of an increase in decomposition rate with temperature when labile carbon is considered. However, for carbon of more recalcitrant types with long turnover times (years to centuries) which make up the majority of the carbon found in soil, the absolute temperature induced changes are small, although the relative sensitivity is generally found to be higher than in the labile carbon fraction [7, 8]. Further, processes of adsorption and desorption as well as aggregate turnover, respond to temperature, and affect the availability of carbon to the decomposition process [8]. The observation that soil warming experiments have most often reported a return to pre-warming levels of carbon efflux after a few years of elevated fluxes have been explained by the decomposition response of the most labile soil carbon fractions [67, 68], but it has also been suggested that the apparent stabilization on pre-warming levels is a result of microbial adaptation and physiology [69]. Large uncertainty exists on the anticipated changes in the more recalcitrant part of the soil carbon stock [8, 70], and the drivers of change are likely to go far beyond the effects of temperature and precipitation which were the main drivers in the current study. Further, plant functional traits may influence soil carbon dynamics in ways not yet fully understood [71–73]. On a global scale, whether or not carbon input to the soil from vegetation is larger than decomposition is unresolved [7].

In the current methodology used for UNFCCC reporting, relying on NFI registrations ensures that any effects on tree growth resulting from environmental changes in, for example, CO_2_-concentration, temperature [74], precipitation [75], and N-deposition [5] is included in the biomass estimates to the extent this is reflected in biomass models. A gradually increasing litter input as used in this study is expected to result from environmental changes in addition to an effect from the general increase in forest age across the Norwegian forest area due to a relatively low harvest rate. However, using the long-term average climate to estimate decomposition and soil carbon changes, (e.g. [44]), in a period where temperature (and to some extent also precipitation) has increased, means that estimates of litter input and decomposition to some extent are decoupled. Following this argument, the soil carbon change estimate based on the long-term average climate is directly coupled to any possible changes in litter production, but not to possible changes in decomposition rate. The mean annual national total soil carbon change was approximately 900–1000 Gg C larger when using constant climatic input compared to using annual or 5-year means–this is equivalent to 13–15% of the annual carbon changes estimated in the living biomass [44]. When the variability in climatic input was considered (this study), change estimates for soil carbon fall outside the confidence interval calculated for soil carbon changes in Norway’s official UNFCCC reporting (15%, 2 standard errors) [44].

It must be expected that the magnitude of the effects of temporal aggregation will depend on the length of the period of aggregation and the strength of the climatic trend. For all three levels of temporal aggregation, the national level soil carbon changes were generally positive with an increasing trend for the period relevant to the UNFCCC reporting. Uncertainties are generally large for estimates of soil carbon changes [28]. The sensitivity of soil carbon change estimates to temporal aggregation of input data was documented in a Finnish study where annual weather data were used as input to Yasso07. Estimates of mean annual soil carbon changes resulted in a 40% lower estimate than if long-term mean weather was applied [24]. The changes were particularly sensitive to the variability in precipitation. In warm years with high precipitation, the overall soil carbon balance turned from a sink (mean weather) to a source (annual weather). The ROMUL model–applied to the same dataset [24]–showed the opposite response, with the highest change estimate for annual weather data and a 50% lower soil carbon change estimate when using mean weather. The ROMUL model is capable of simulating the effects of extreme soil climatic conditions (daily time scale, drought and water logging) on decomposition. Thus, when using climatic means over long time periods, these effects are excluded and this is the reason behind the different behavior of the two models in the Finnish study. All model estimates in the Finnish study were within the bounds of measurement-based estimates of soil carbon change (in the organic layer of upland forest). Thus, comparing to measured changes could not resolve which temporal resolution was better [24]. National scale measurements of forest soil carbon changes are not available for Norway.

The magnitude of our soil carbon change estimates generally conformed to measured results in Scandinavian studies. Median of the model estimated soil carbon change for coniferous forest in southeast and northern Norway for age-classes 40–80 years was 20–30 g C m^-2^ year^-1^ when 5-year average climate was used. This compares to 23 (+/-2) g C m^-2^ year^-1^ measured in the organic layer of boreal forests in Finland of similar age-classes [76], a mean sequestration rate of 25 g C m^-2^ year^-1^ in the humus layer of Swedish forest [77], and a mean change of 29 (+/- 31) g C m^-2^ year^-1^ for the Swedish forest soil inventory [32]. In our study, when using a long-term average climate, the median change rate was similar in northern Norway, but as high as 40 g C m^-2^ year^-1^ in the southeastern part of the country. We assume that the forests in the southwestern parts of Norway are profoundly different from the forests of Sweden and Finland and a comparison is not meaningful.

## Conclusions

The results illustrated that, using the soil carbon and decomposition model Yasso07, both spatial aggregation and temporal aggregation of climatic data had considerable effects on the modeled soil carbon results. However, the direction and magnitude of the differences resulting from spatial aggregation will depend on the region of interest. The general conclusion of this study is therefore that the level of aggregation will influence the estimated soil carbon dynamics and a systematic error will be introduced. Secondly, our results illustrated that, in a time with a strong climatic trend, the use of longer-term averages of climatic input data had strong effects on soil carbon estimates. In northern regions with strongly increasing temperatures it must generally be expected that using long-term mean climatic data will result in higher estimated accumulation rates than when the climatic trends are specifically represented at a higher temporal resolution. When modeling soil carbon for scientific and reporting purposes, our results illustrated that caution should be used when interpreting or producing soil carbon estimates, and considerations on spatial and temporal aggregation should be specifically considered.
